# A Dual-Branch Fusion Model for Deepfake Detection Using Video Frames and Microexpression Features

**DOI:** 10.3390/jimaging11070231

**Published:** 2025-07-11

**Authors:** Georgios Petmezas, Vazgken Vanian, Manuel Pastor Rufete, Eleana E. I. Almaloglou, Dimitris Zarpalas

**Affiliations:** 1 Centre for Research and Technology Hellas, 57001 Thessaloniki, Greece; petmezgs@iti.gr (G.P.); e.alma@iti.gr (E.E.I.A.); zarpalas@iti.gr (D.Z.); 2 Herta Security, 08037 Barcelona, Spain; manuel.pastor@hertasecurity.com

**Keywords:** deepfake detection, microexpressions, fusion model, 3D ResNet, transformer

## Abstract

Deepfake detection has become a critical issue due to the rise of synthetic media and its potential for misuse. In this paper, we propose a novel approach to deepfake detection by combining video frame analysis with facial microexpression features. The dual-branch fusion model utilizes a 3D ResNet18 for spatiotemporal feature extraction and a transformer model to capture microexpression patterns, which are difficult to replicate in manipulated content. We evaluate the model on the widely used FaceForensics++ (FF++) dataset and demonstrate that our approach outperforms existing state-of-the-art methods, achieving 99.81% accuracy and a perfect ROC-AUC score of 100%. The proposed method highlights the importance of integrating diverse data sources for deepfake detection, addressing some of the current limitations of existing systems.

## 1. Introduction

The emergence of deepfake technology has created serious concerns about the trustworthiness of digital media [1]. Deepfakes, synthetic media produced using artificial intelligence methods, can alter videos to create highly realistic but fabricated content. These manipulations convincingly depict individuals saying or doing things they never actually did, raising significant implications for various fields, including politics, entertainment, and personal privacy. Consequently, the development of effective deepfake detection methods has become a crucial priority.

In response to the growing threat of deepfakes, researchers have developed automated detection methods that primarily analyze visual and temporal inconsistencies in video frames [2]. These approaches often rely on convolutional neural networks or other deep learning (DL) architectures to identify artifacts or unnatural patterns, such as mismatched lip-syncing or irregular facial movements. However, as deepfake technology continues to improve, generating increasingly realistic facial appearances and expressions, existing approaches struggle to detect subtle manipulations [3]. Moreover, detecting temporal inconsistencies often requires high-quality data and sophisticated models capable of processing long video sequences, limiting their robustness in diverse real-world scenarios.

This paper proposes a novel approach to deepfake detection by combining traditional video frame analysis with facial microexpression features. Microexpressions are innate, brief facial movements that occur in response to emotions and typically last for less than a second [4]. These rapid movements can reveal underlying facial behaviors that are difficult to replicate convincingly in deepfakes, offering a hidden layer of information that has not been fully exploited in the existing literature. To leverage these features, our methodology employs a two-branch fusion model that integrates two distinct yet complementary types of data, namely (i) raw video frames analyzed through a 3D ResNet18 model and (ii) facial microexpressions processed through a transformer model. By combining these modalities, we aim to create a more robust deepfake detection system that can better handle the challenges posed by increasingly sophisticated deepfake technologies.

The key contributions of this study are as follows:

- We propose a novel dual-branch framework for deepfake detection that combines raw video frame analysis with facial microexpression modeling.
- Unlike previous methods that rely solely on visual artifacts, our approach captures subtle, involuntary facial dynamics by encoding non-rigid shape deformations and emotion-driven action units (AUs) using a temporal transformer.
- We introduce a modality-aware fusion mechanism, enhancing the model’s sensitivity to both visual and behavioral inconsistencies typically overlooked by deepfake generators.
- The proposed approach outperforms state-of-the-art methods on the FF++ dataset, validating its effectiveness and setting a new benchmark for deepfake detection.

The remainder of this paper is structured as follows: In Section 2, we review existing video deepfake detection methods. Section 3 outlines the research methodology in detail. In Section 4, we evaluate the performance of the proposed model, while Section 5 interprets the research findings and highlights open research problems. Finally, in Section 6, the conclusions of this study are summarized.

## 2. Related Work

Deepfake detection often leverages DL to uncover subtle artifacts across spatial, frequency, and temporal domains. For instance, Zhang et al. [5] introduced a two-stream architecture with a multiscale transformer module and a fusion mechanism to analyze spatial and noise flow artifacts. Similarly, Miao et al. [6] proposed F2Trans, which combines wavelet sampling and central difference attention for frequency-aware feature extraction. Zhang et al. [7] used dual-domain fusion and local enhancement attention to enrich feature representation.

Several recent studies have adopted dual-branch structures to balance local detail and global context. Guo et al. [8] introduced LDFNet, a lightweight detector that fuses local visual artifacts and global texture cues via a dynamic fusion module and TraceBlock-based inference. Long et al. [9] proposed LGDF-Net, which explicitly separates and processes local and global features using specialized modules and fuses them through a multi-level strategy.

Other methods focus on temporal or textural robustness. Pang et al. [10] captured spatiotemporal dependencies through a multi-rate excitation network. Yang et al. [11] and Zhao et al. [12] emphasized texture-based cues using attention mechanisms and feature enhancement blocks.

In a related direction, Cheng et al. [13] proposed leveraging 3D facial geometry by measuring inconsistencies between 2D landmarks and 3D reprojections across video frames, capturing subtle temporal anomalies using an RNN-based framework. Meanwhile, Wang et al. [14] introduced WATCHER, which employs wavelet-guided hierarchical learning to jointly reason over texture and content features through multi-domain fusion and attention mechanisms, achieving superior generalization to unseen manipulations.

In parallel, generalization and robustness across datasets have emerged as pressing concerns. Alfalasi et al. [15] addressed this by evaluating detection methods under novel deepfake types and proposed randomized synthetic benchmarks for more reliable cross-dataset performance evaluation.

While these studies have significantly advanced deepfake detection by leveraging spatial, frequency, and texture-based features, a notable gap in the literature remains, namely the incorporation of facial dynamics such as microexpressions, which are critical in distinguishing real and fake videos. Our study bridges this gap by focusing on these overlooked features, offering a novel perspective that complements existing approaches and enhances detection robustness.

## 3. Methodology

### 3.1. Datasets

For our experiments, we utilized two widely recognized deepfake detection benchmarks, namely FF++ [16] and Celeb-DF [17], as well as a recently introduced deepfake dataset named ReenactFaces [18].

FF++ is a comprehensive dataset comprising 1000 original video sequences sourced from 977 YouTube videos, primarily featuring frontal unobstructed faces suitable for manipulation. These original videos are altered using four advanced techniques, including (i) DeepFakes; (ii) Face2Face; (iii) FaceSwap; and (iv) NeuralTextures, resulting in 4000 forged videos. In this study, we followed the official split, with 3600 videos for training, 700 for validation, and 700 for testing.

To evaluate the generalizability of our approach, we extended our experiments to Celeb-DF, which includes 590 high-quality original YouTube videos featuring 59 celebrities, alongside 5639 deepfake videos. These forgeries are created using an improved synthesis method that produces more photorealistic manipulations than earlier datasets. The dataset introduces additional challenges due to its high video quality, demographic diversity, and subtle artifacts, making it an ideal benchmark for assessing detection robustness across real-world scenarios.

We further benchmarked our method on ReenactFaces, a new dataset comprising 10,000 real and 10,000 re-enacted videos created using three state-of-the-art facial re-enactment methods, namely the First-Order Motion Model [19], Thin-Plate Spline Motion [20], and FSRT [21]. Videos were sourced from YouTube to ensure demographic diversity and realistic speaking behavior, and the manipulations focus solely on facial re-enactment rather than face swapping. Compared to existing benchmarks, ReenactFaces provides more natural motion patterns and poses a unique challenge due to the subtle and temporally coherent nature of its manipulations.

### 3.2. Preprocessing

To prepare the data for training, validation, and testing, we applied a comprehensive preprocessing pipeline to the datasets. For video preprocessing, individual video frames were extracted, followed by facial cropping and vertical alignment of each frame to ensure consistent input dimensions and alignment across the entire dataset. The cropped frames were then resized to 112 × 112 pixels, a common input size for face recognition tasks. Next, the pixel values of the frames were normalized using a common standardization process by centering the data around a mean of 0 and scaling it based on the standard deviation, making it suitable for DL models. Finally, to capture temporal dynamics in videos, the preprocessed frames were organized into sequences of 96 consecutive frames. This sequence length was chosen to balance computational efficiency and the need to provide sufficient temporal context for the proposed DL model.

### 3.3. Microexpression Extraction

**Figure 1:**
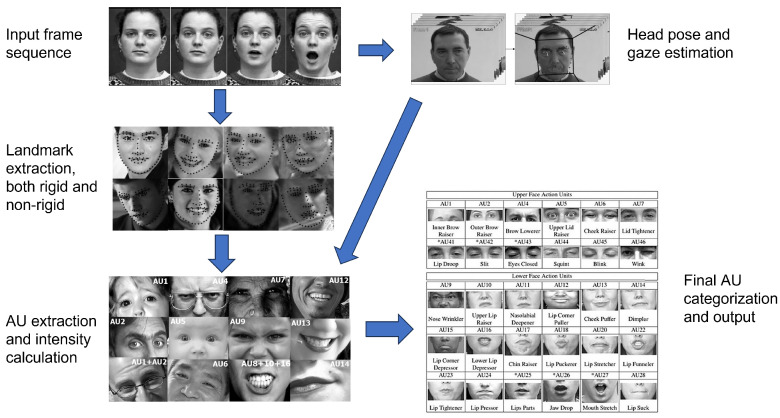
The detailed microexpression extraction pipeline. It begins with an input facial frame sequence, which is processed through two parallel paths. In the first path, facial landmark detection is performed, where black dots indicate the extracted 2D facial landmarks, including both rigid and non-rigid components. In the second path, the same sequence is used for head pose and gaze estimation. Both streams then feed into a shared module for AU extraction and intensity estimation, culminating in the final AU categorization. Arrows denote the directional flow of data between processing stages.

Microexpressions can offer valuable information for deepfake detection due to their innate and involuntary nature, which is challenging to replicate in manipulated videos. To leverage this, we followed the methodology by Baltrušaitis et al. [22] to perform a feature-based analysis of facial landmarks and AUs, extracting the relevant microexpression features from the preprocessed video frames. Non-rigid face shape parameters were computed to capture facial deformations arising from expressions and identity—each parameter represents a 2D facial landmark point. Additionally, facial AUs were analyzed to provide complementary information about expression intensity and presence. AUs correspond to anatomically based facial muscle movements defined by the Facial Action Coding System (FACS) [23], representing fundamental units of facial expressions. Each AU indicates a specific facial muscle contraction or movement, which together characterize complex expressions. In this study, the intensity (on a scale of 0 to 5) was extracted for 17 AUs to quantify the strength of facial movements during the expression, while the presence (binary 0 or 1) of 18 AUs was detected to identify whether certain facial movements occurred during the expression. In total, 69 features were extracted for each sequence of 96 frames to form the input to the transformer-based branch of our model. In this way, the temporal context—how those features change over time in a video—was also taken into account in the analysis. A diagram illustrating the detailed microexpression extraction pipeline is presented in Figure 1, while a complete list of the extracted parameters is provided in Table A1 and Table A2.

### 3.4. DL Models

The proposed system employed two widely used DL architectures, including (i) a 3D ResNet18 and (ii) a transformer model. The 3D ResNet18 [24] extends the ResNet architecture to video data by incorporating 3D convolutions, enabling it to learn spatiotemporal features from sequences of frames. It is commonly applied in video classification and action recognition tasks due to its effectiveness in modeling temporal dynamics in video data. On the other hand, transformer [25] models, originally introduced for natural language processing, have gained prominence in vision tasks due to their ability to model long-range dependencies and relationships. They are widely used in video understanding and multimodal learning for their attention mechanism, which excels at capturing complex patterns in sequential data. The specific implementation and integration of these models within our framework are detailed in Section 3.5.

### 3.5. Fusion Approach

**Figure 2:**
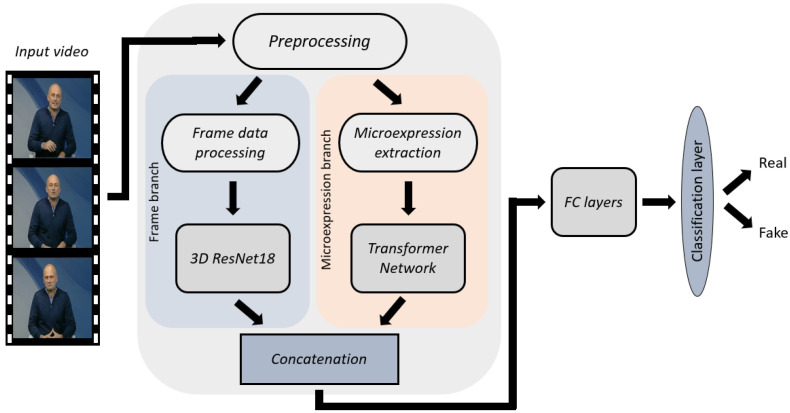
Overview of the proposed methodology.

**Figure 3:**
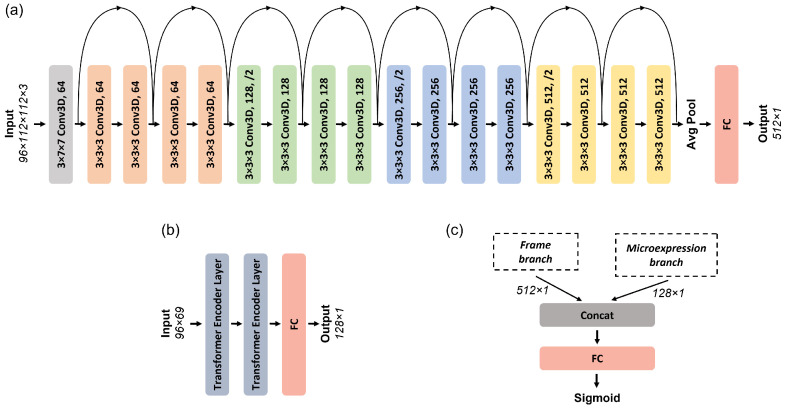
Detailed architecture of the dual-branch fusion model, illustrating (**a**) the 3D ResNet18-based frame branch, (**b**) the Transformer-based microexpression branch, and (**c**) the late fusion and classification stage.

To leverage both spatiotemporal and microexpression modalities, we implemented a dual-branch fusion approach. An overview of the proposed approach is presented in Figure 2, while a more detailed view of the architecture of each branch and their fusion is illustrated in Figure 3.

The first branch employed a 3D ResNet18 architecture, pre-trained on Kinetics-400 [26], a large-scale video dataset designed to capture diverse human actions in various contexts. This network comprises 18 convolutional layers structured into four residual blocks, each with two 3D convolutional layers, followed by batch normalization and ReLU activation. The input to the model consists of sequences of 96 RGB frames resized to 112 × 112 pixels. For our task, the pre-trained model was fine-tuned to capture spatiotemporal patterns specific to deepfake detection. The final fully connected (FC) layer of the original model was removed, and the output from the last global average pooling layer was used to obtain a 512-dimensional feature vector per sequence.

The second branch utilized a lightweight transformer model tailored for sequential microexpression data. It consisted of two encoder layers, each containing multi-head self-attention with three heads, a model dimensionality of 128, and a feedforward network of size 256. Positional encodings were added to the input to preserve temporal ordering. This branch processed sequences of 96 time steps, where each time step consisted of a 69-dimensional vector formed by concatenating non-rigid facial shape parameters and AU features (as described in Section 3.3). The final output was passed through an FC layer that reduced the representation to 128 dimensions.

The outputs of the 3D ResNet18 (512-dimensional) and transformer (128-dimensional) branches were then concatenated to form a 640-dimensional combined feature vector. We adopted feature-level fusion due to its simplicity, computational efficiency, and strong empirical performance. This approach allows the model to integrate complementary cues from both modalities early in the decision process while avoiding the complexity and overhead of decision-level or attention-based fusion strategies. This joint representation was passed through a classification head composed of two FC layers: the first reduced the vector to 128 units with ReLU activation, followed by a second layer projecting to a single-output neuron with a sigmoid activation for binary classification (real vs. fake). This strategy was critical for improving the model’s ability to distinguish between authentic and manipulated videos, particularly in cases where spatial or temporal inconsistencies alone were insufficient for detection.

## 4. Results

### 4.1. Experimental Setup

The DL model for detecting video deepfakes was developed and tested in a Python 3.9 environment, with PyTorch (version 2.4.1) serving as the main framework for model implementation. All computational tasks were carried out on a workstation equipped with an NVIDIA GeForce RTX 3060 GPU and utilizing CUDA 11.6, which enabled efficient processing for the demanding training and evaluation phases.

The model was trained using a binary cross-entropy loss function, optimized with the Adam optimizer (learning rate = $1\times 10^{-4}$) and a cosine annealing learning rate scheduler. Training was performed over 100 epochs with early stopping triggered if the validation loss did not improve for 10 consecutive epochs. A batch size of eight was used to accommodate the computational requirements of processing both video frames and microexpression features. The model’s performance was evaluated on the test set using accuracy, precision, recall, F1 score, and area under the receiver operating characteristic curve (ROC-AUC).

### 4.2. Experimental Evaluation

To evaluate the contribution of each modality to the overall performance of our model, we conducted an ablation study. We tested the model’s performance using three different configurations as follows: (i) using only the frame branch; (ii) using only the microexpression branch; and (iii) combining both modalities in the fusion model.

As shown in Table 1, the fusion model, which integrates both video frames and microexpression features, achieved the highest performance across all metrics on the FF++ dataset. Specifically, it demonstrated exceptional accuracy (99.81%), precision (99.88%), recall (99.88%), and F1 score (99.88%), with a perfect ROC-AUC score of 100%. These results highlight the significant advantage of combining spatiotemporal features from video frames with microexpression patterns, enabling the model to more effectively detect deepfakes. The frame-only branch also performed well with high accuracy and precision, but the fusion consistently outperformed both individual branches. This underscores the complementary nature of microexpressions and temporal context in robust deepfake detection.

To further investigate the impact of the transformer configuration in our fusion model, we performed an ablation study varying the number of encoder layers and attention heads. As shown in Table 2, using two encoder layers and three attention heads resulted in the best performance, achieving an accuracy of 99.81%, F1 score of 99.88%, and perfect ROC-AUC of 100%. Reducing the encoder layers to one or increasing them to three led to slight performance degradation, likely due to underfitting or overfitting, respectively. Similarly, adjusting the number of attention heads from 3 to either 1 or 23—both divisors of the input embedding dimension of the microexpression features (69)—also resulted in reduced performance. This is consistent with the notion that an intermediate number of heads strikes a better balance between representation capacity and training stability. These results validate that our chosen configuration effectively balances model complexity and generalization, confirming the optimality of the transformer structure used in the proposed fusion model.

In addition to evaluation metrics, we assessed the inference efficiency of each model variant. The average frame processing rates were 13 FPS for the frame branch, 15 FPS for the microexpression branch, and 12 FPS for the fusion model. Despite integrating both modalities, the fusion model maintains a competitive inference speed close to real time, highlighting its practicality for deployment in realistic settings. These results indicate that our approach effectively balances high detection accuracy with computational efficiency.

To test the generalizability of our approach, we evaluated the same models on the Celeb-DF dataset [17]. As shown in Table 3, the fusion model once again achieved perfect scores across all metrics, confirming its effectiveness on more challenging high-fidelity forgeries. Interestingly, the frame branch alone also performed strongly (99.78% accuracy and 100% ROC-AUC), while the microexpression branch showed a notable drop in precision but maintained high recall—highlighting its sensitivity to manipulated cues, albeit with more false positives. The fusion of both branches balanced these strengths to deliver unmatched detection reliability. It is also worth noting that although the model was trained exclusively on FF++, its robust learned features generalize well to Celeb-DF’s distinct forgery characteristics, enabling superior detection performance even without direct training on this dataset. These results demonstrate that the proposed model maintains state-of-the-art performance not only on FF++ but also on Celeb-DF, affirming its robustness across datasets with different characteristics and difficulty levels.

Furthermore, we conducted additional experiments on the ReenactFaces dataset [18], a specialized open access dataset focusing on facial re-enactment manipulations. ReenactFaces offers a targeted benchmark for reenactment deepfakes, addressing a critical gap in current datasets, which often emphasize single manipulation types. It comprises both real and re-enacted videos, enabling a precise evaluation of models’ robustness to re-enactment-based deepfakes, which pose unique detection challenges due to subtle facial motion and expression transfer.

The experimental results are summarized in Table 4. Our fusion model again achieves superior performance, with accuracy reaching 99.13%, a precision of 99.59%, recall of 98.78%, F1 score of 98.65%, and an outstanding ROC-AUC of 99.71%. The frame-only branch also performed well, confirming the effectiveness of spatiotemporal features, while the microexpression branch showed comparatively lower recall, indicating some difficulty capturing re-enactment-specific cues independently. Nonetheless, the fusion of both modalities demonstrates strong complementarity and robust detection capability.

These findings demonstrate that our proposed fusion model generalizes effectively beyond common deepfake datasets, including challenging re-enactment manipulations. This supports the model’s applicability for real-world scenarios where diverse forgery techniques may be encountered.

### 4.3. Comparison with State-of-the-Art Methods

To further assess the effectiveness of our proposed approach, we compared its performance with several state-of-the-art deepfake detection methods. These include both image-based and video-based approaches, which operate on different input modalities but are commonly benchmarked on FF++. The results of this comparison are presented in Table 5. Note that the performance metrics of the other methods are those reported in the respective papers, where the authors followed consistent training and evaluation procedures using the FF++ dataset, identical to those used in this study. Comparative results for Celeb-DF and ReenactFaces are not included, as consistent training and evaluation protocols or official implementations for all referenced methods are not publicly available, making such comparisons infeasible.

The proposed model significantly outperforms all the compared methods in terms of both accuracy (99.81%) and ROC-AUC (100%). While the best-performing method in the literature by Miao et al. [6] achieved an accuracy of 98.71% and an ROC-AUC of 99.74%, our model improves upon this by over one percentage point in accuracy and achieves a perfect ROC-AUC. Other methods such as those by Long et al. [9] and Yang et al. [11] performed well but were still significantly below our model’s performance.

This performance improvement can be attributed to the fusion of both spatiotemporal and microexpression features, which together provide a richer representation that enhances the model’s ability to detect even the most subtle manipulations in deepfake videos.

## 5. Discussion

This study demonstrated a novel approach to deepfake detection by fusing spatiotemporal video frame features with microexpression-based features. The results showcase the effectiveness of leveraging complementary modalities, achieving state-of-the-art performance on the FF++ dataset. However, while the proposed method shows great promise, the larger context of deepfake detection requires ongoing efforts to create robust systems capable of addressing the wide variety of deepfake manipulation techniques and their evolving nature.

Despite considerable advancements, there are several open research problems in the domain of deepfake detection. One of the key challenges is the constant evolution of deepfake generation techniques, which increasingly focus on improving realism and reducing detectable artifacts. As deepfake models improve, the corresponding detection models must be equally adaptive. Current methods are often limited to detecting specific forms of manipulation and may not generalize well to new or unseen types of deepfakes [27].

Furthermore, the need for high-quality data and sophisticated models to analyze long video sequences presents another barrier, as many existing methods struggle with scalability or require significant computational resources. Additionally, while many methods focus on visual and spatiotemporal features, there is still a lack of comprehensive systems that combine multiple forms of information, such as microexpressions, audio, and motion, to enhance robustness across a broader range of manipulations.

This study addresses some of these challenges by incorporating facial microexpression features—involuntary facial movements that are difficult to replicate—alongside spatiotemporal video frame analysis. To the best of our knowledge, this is the first time microexpressions have been utilized for deepfake detection, marking a significant advancement in the field. The proposed dual-branch fusion model significantly outperforms existing state-of-the-art methods when evaluated on the FF++ dataset. This highlights the importance of integrating complementary features to detect even the most refined deepfake manipulations and presents a new avenue for improving detection systems in future research.

While the proposed model shows promising results, there are a few limitations. First, its evaluation was limited to three deepfake datasets, which may not fully capture the diversity of new deepfake techniques. Moreover, while facial microexpressions improve detection, other modalities like audio and motion analysis remain underexplored. Also, while the extracted AU and shape features are generally robust, their reliability may be affected under less ideal conditions such as partial occlusions (i.e., glasses or hands), side-profile views, or suboptimal lighting. Furthermore, although the datasets used in this study do contain various forms of compression and natural variations, they do not fully replicate the range of visual distortions common in real-world media, such as social media filters, watermarks, or postproduction effects. Evaluating the model’s robustness under these conditions remains an important direction for future work to better understand its applicability in unconstrained environments. Although the deployed datasets may already include such variations to some extent, we have not explicitly assessed their impact, and further validation would be necessary to fully understand model behavior in these scenarios. Finally, although the model achieves strong performance, its interpretability is currently limited, which could be a barrier to adoption in high-stakes domains. In future work, we aim to test the model on a wider range of datasets, incorporate additional modalities, explore more advanced fusion strategies, and investigate interpretability tools such as attention visualization to enhance model explainability and user trust.

## 6. Conclusions

In this study, we proposed a novel deepfake detection method that fuses spatiotemporal video frame analysis with facial microexpression features. This dual-branch approach achieved state-of-the-art results on the FF++ dataset, highlighting the potential of leveraging involuntary facial movements to enhance detection robustness. While promising, ongoing research is necessary to adapt to evolving synthesis techniques. Future work should explore additional modalities and more diverse datasets to further improve generalizability and investigate novel approaches to enhance interpretability.

## Figures and Tables

**Table 1:** Results of the ablation study on the FF++ dataset, showing the performance of the model using only the frame branch, only the microexpression branch, and the fusion of both modalities. The best value for each metric is highlighted in bold.

Method	Accuracy	Precision	Recall	F1 Score	ROC-AUC
Frame branch	98.69%	99.38%	98.95%	99.16%	99.91%
Microexpression branch	84.68%	89.50%	91.10%	90.29%	86.30%
**Fusion (proposed)**	**99.81%**	**99.88%**	**99.88%**	**99.88%**	**100%**

**Table 2:** Ablation study on the number of transformer encoder layers and attention heads in the fusion model. The best value for each metric is highlighted in bold.

Layers	Heads	Accuracy	F1 Score	ROC-AUC
1	3	98.92%	99.05%	99.24%
**2**	**3**	**99.81%**	**99.88%**	**100%**
3	3	99.09%	99.23%	99.47%
2	1	98.65%	98.75%	98.88%
2	23	98.34%	98.42%	98.49%

**Table 3:** Results of the ablation study on the Celeb-DF dataset, showing the performance of the model using only the frame branch, only the microexpression branch, and the fusion of both modalities. The best value for each metric is highlighted in bold.

Method	Accuracy	Precision	Recall	F1 Score	ROC-AUC
Frame branch	99.78%	99.74%	99.91%	99.83%	**100%**
Microexpression branch	77.40%	74.83%	98.01%	84.87%	87.57%
**Fusion (proposed)**	**100%**	**100%**	**100%**	**100%**	**100%**

**Table 4:** Results of the ablation study on the ReenactFaces dataset, showing the performance of the model using only the frame branch, only the microexpression branch, and the fusion of both modalities. The best value for each metric is highlighted in bold.

Method	Accuracy	Precision	Recall	F1 Score	ROC-AUC
Frame branch	96.21%	96.85%	96.26%	96.56%	96.54%
Microexpression branch	85.85%	95.82%	80.80%	87.67%	96.26%
**Fusion (proposed)**	**99.13%**	**99.59%**	**98.78%**	**98.65%**	**99.71%**

**Table 5:** Comparison with state-of-the-art deepfake detection methods on the FF++ dataset. The best value for each metric is highlighted in bold.

Method	Accuracy	ROC-AUC
Zhang et al. [5]	95.20%	98.68%
Miao et al. [6]	98.71%	99.74%
Zhang et al. [7]	94.14%	98.44%
Guo et al. [8]	96.01%	98.92%
Long et al. [9]	97.64%	99.70%
Pang et al. [10]	97.76%	98.81%
Yang et al. [11]	97.86%	99.38%
Zhao et al. [12]	97.60%	99.29%
Alfalasi et al. [15]	97.31%	99.60%
**Ours**	**99.81%**	**100%**

## Data Availability

The datasets used and analyzed during the present study are open access and can be found as follows: the FF++ dataset is available through https://github.com/ondyari/FaceForensics (accessed on 7 July 2025), the Celeb-DF dataset is available through https://github.com/yuezunli/celeb-deepfakeforensics (accessed on 7 July 2025), while the ReenactFaces dataset is available through https://zenodo.org/records/14035828 (accessed on 7 July 2025).

## Appendix A

**Table A1:** Detailed list of non-rigid shape parameters grouped by facial regions.

Parameters	Facial Region
0–1	Right cheek, upper part
2–4	Right cheek, middle part
5–6	Right cheek, lower part
7–9	Chin
10–11	Left cheek, lower part
12–14	Left cheek, middle part
15–16	Left cheek, upper part
17–21	Right eyebrow
22–26	Left eyebrow
27–30	Nose, vertical
31–35	Nose, horizontal

**Table A2:** Detailed list of facial action units (AUs) with presence and intensity indicators.

AU	Definition	Presence	Intensity
1	Inner Brow Raiser	✓	✓
2	Outer Brow Raiser	✓	✓
3	Brow Lowerer	✓	✓
4	Upper Lid Raiser	✓	✓
5	Cheek Raiser	✓	✓
6	Lid Tightener	✓	✓
7	Nose Wrinkler	✓	✓
8	Upper Lip Raiser	✓	✓
9	Lip Corner Puller	✓	✓
10	Dimpler	✓	✓
11	Lip Corner Depressor	✓	✓
12	Chin Raiser	✓	✓
13	Lip Stretcher	✓	✓
14	Lip Tightener	✓	✓
15	Lips Part	✓	✓
16	Jaw Drop	✓	✓
17	Lip Suck	✓	×
18	Blink	✓	✓

## References

[1] Rana M.S., Nobi M.N., Murali B., Sung A.H. (2022). Deepfake Detection: A Systematic Literature Review. IEEE Access.

[2] Heidari A., Navimipour N.J., Dag H., Unal M. (2023). Deepfake detection using deep learning methods: A systematic and comprehensive review. Wiley Interdiscip. Rev. Data Min. Knowl. Discov..

[3] Kaur A., Hoshyar A.N., Saikrishna V., Firmin S., Xia F. (2024). Deepfake video detection: Challenges and opportunities. Artif. Intell. Rev..

[4] Xie H., Lo L., Shuai H.-H., Cheng W.-H. (2020). An Overview of Facial Micro-Expression Analysis: Data, Methodology and Challenge. IEEE Trans. Affect. Comput..

[5] Zhang D., He R., Liao X., Li F., Chen J., Yang G. (2024). Face Forgery Detection Based on Fine-grained Clues and Noise Inconsistency. IEEE Trans. Artif. Intell..

[6] Miao C., Tan Z., Chu Q., Liu H., Hu H., Yu N. (2023). F2Trans: High-Frequency Fine-Grained Transformer for Face Forgery Detection. IEEE Trans. Inf. Forensics Secur..

[7] Zhang D., Chen J., Liao X., Li F., Chen J., Yang G. (2024). Face Forgery Detection via Multi-Feature Fusion and Local Enhancement. IEEE Trans. Circuits Syst. Video Technol..

[8] Guo Z., Wang L., Yang W., Yang G., Li K. (2024). LDFnet: Lightweight Dynamic Fusion Network for Face Forgery Detection by Integrating Local Artifacts and Global Texture Information. IEEE Trans. Circuits Syst. Video Technol..

[9] Long M., Liu Z., Zhang L.-B., Peng F. (2025). LGDF-Net: Local and Global Feature Based Dual-Branch Fusion Networks for Deepfake Detection. IEEE Trans. Circuits Syst. Video Technol..

[10] Pang G., Zhang B., Teng Z., Qi Z., Fan J. (2023). MRE-Net: Multi-Rate Excitation Network for Deepfake Video Detection. IEEE Trans. Circuits Syst. Video Technol..

[11] Yang J., Li A., Xiao S., Lu W., Gao X. (2021). MTD-Net: Learning to Detect Deepfakes Images by Multi-Scale Texture Difference. IEEE Trans. Inf. Forensics Secur..

[12] Zhao H., Zhou W., Chen D., Wei T., Zhang W., Yu N. Multi-attentional Deepfake Detection. Proceedings of the 2021 IEEE/CVF Conference on Computer Vision and Pattern Recognition (CVPR).

[13] Cheng Z., Chen C., Zhou Y., Hu X. Mining Temporal Inconsistency with 3D Face Model for Deepfake Video Detection. Proceedings of the Chinese Conference on Pattern Recognition and Computer Vision.

[14] Wang Y., Chen C., Zhang N., Hu X. (2024). WATCHER: Wavelet-Guided Texture-Content Hierarchical Relation Learning for Deepfake Detection. Int. J. Comput. Vis..

[15] Alfalasi H.R., Hashem I.A., Abul O. Generalizable Spatiotemporal Deepfake Detection with Late-Fusion. Proceedings of the 2024 7th International Conference on Signal Processing and Information Security (ICSPIS).

[16] Rössler A., Cozzolino D., Verdoliva L., Riess C., Thies J., Nießner M. FaceForensics++: Learning to Detect Manipulated Facial Images. Proceedings of the 2019 IEEE/CVF International Conference on Computer Vision (ICCV).

[17] Li Y., Yang X., Sun P., Qi H., Lyu S. Celeb-DF: A Large-Scale Challenging Dataset for DeepFake Forensics. Proceedings of the 2020 IEEE/CVF Conference on Computer Vision and Pattern Recognition (CVPR).

[18] Vanian V., Petmezas G., Konstantoudakis K., Zarpalas D. (2025). ReenactFaces: A Specialized Dataset for Reenactment-Based Deepfake Detection. International Conference on Advanced Information Networking and Applications.

[19] Siarohin A., Woodford O.J., Ren J., Chai M., Tulyakov S. Motion Representations for Articulated Animation. Proceedings of the IEEE/CVF Conference on Computer Vision and Pattern Recognition (CVPR).

[20] Zhao J., Zhang H. Thin-Plate Spline Motion Model for Image Animation. Proceedings of the IEEE/CVF Conference on Computer Vision and Pattern Recognition (CVPR).

[21] Rochow A., Schwarz M., Behnke S. FSRT: Facial Scene Representation Transformer for Face Reenactment from Factorized Appearance, Head-Pose, and Facial Expression Features. Proceedings of the IEEE/CVF Conference on Computer Vision and Pattern Recognition (CVPR).

[22] Baltrušaitis T., Mahmoud M.M., Robinson P. Cross-dataset learning and person-specific normalisation for automatic Action Unit detection. Proceedings of the 2015 11th IEEE International Conference and Workshops on Automatic Face and Gesture Recognition (FG).

[23] Ekman P., Friesen W.V. (1978). Facial Action Coding System: A Technique for the Measurement of Facial Movement.

[24] Hara K., Kataoka H., Satoh Y. Learning Spatio-Temporal Features with 3D Residual Networks for Action Recognition. Proceedings of the 2017 IEEE International Conference on Computer Vision Workshops (ICCVW).

[25] Vaswani A., Shazeer N.M., Parmar N., Uszkoreit J., Jones L., Gomez A.N., Kaiser L., Polosukhin I. Attention is All you Need. Proceedings of the 31st Conference on Neural Information Processing Systems (NeurIPS).

[26] Kay W., Carreira J., Simonyan K., Zhang B., Hillier C., Vijayanarasimhan S., Viola F., Green T., Back T., Natsev A. (2017). The Kinetics Human Action Video Dataset. arXiv.

[27] Petmezas G., Vanian V., Konstantoudakis K., Almaloglou E.E.I., Zarpalas D. (2025). Video deepfake detection using a hybrid CNN-LSTM-Transformer model for identity verification. Multimed. Tools Appl..

